# NANOG and LIN28 dramatically improve human cell reprogramming by modulating LIN41 and canonical WNT activities

**DOI:** 10.1242/bio.047225

**Published:** 2019-12-05

**Authors:** Ling Wang, Yue Su, Chang Huang, Yexuan Yin, Alexander Chu, Alec Knupp, Young Tang

**Affiliations:** Department of Animal Science, Institute for Systems Genomics, University of Connecticut, 1390 Storrs Rd, Storrs, CT 06269, USA

**Keywords:** Induced pluripotent stem cell (iPSC), LIN28, LIN41, WNT, Epithelialization, Reprogramming efficiency

## Abstract

Human cell reprogramming remains extremely inefficient and the underlying mechanisms by different reprogramming factors are elusive. We found that NANOG and LIN28 (NL) synergize to improve OCT4, SOX2, KLF4 and MYC (OSKM)-mediated reprogramming by ∼76-fold and shorten reprogramming latency by at least 1 week. This synergy is inhibited by GLIS1 but reinforced by an inhibitor of the histone methyltransferase DOT1L (iDOT1L) to a ∼127-fold increase in TRA-1-60-positive (+) iPSC colonies. Mechanistically, NL serve as the main drivers of reprogramming in cell epithelialization, the expression of Let-7 miRNA target *LIN41*, and the activation of canonical WNT/β-CATENIN signaling, which can be further enhanced by iDOT1L treatment. *LIN41* overexpression in addition to OSKM similarly promoted cell epithelialization and WNT activation in reprogramming, and a dominant-negative LIN41 mutation significantly blocked NL- and iDOT1L-enhanced reprogramming. We also found that NL- and iDOT1L-induced canonical WNT activation facilitates the initial development kinetics of iPSCs. However, a substantial increase in more mature, homogeneous TRA-1-60+ colony formation was achieved by inhibiting WNT activity at the middle-to-late-reprogramming stage. We further found that LIN41 can replace LIN28 to synergize with NANOG, and that the coexpression of LIN41 with NL further enhanced the formation of mature iPSCs under WNT inhibition. Our study established LIN41 and canonical WNT signaling as the key downstream effectors of NL for the dramatic improvement in reprogramming efficiency and kinetics, and optimized a condition for the robust formation of mature human iPSC colonies from primary cells.

This article has an associated First Person interview with the first author of the paper.

## Abstract (precis)

**Summary:** Robust human iPSC reprogramming through the synergy of NANOG, LIN28, and inhibition of DOT1L in modulation of LIN41 expression and the canonical WNT pathway.

## INTRODUCTION

Two gene cocktails, *OCT4*, *SOX2*, *KLF4*, and *MYC* (OSKM) (Takahashi et al., 2007; Takahashi and Yamanaka, 2006) and *OCT4*, *SOX2*, *NANOG* and *LIN28A* (OSNL) (Yu et al., 2007b), can reprogram somatic cells to embryonic stem cell (ESC)-like induced pluripotent stem cells (iPSCs). The reprogramming of mouse somatic cells involves two major waves of transcriptional changes (Hussein et al., 2014). The first transcriptional change occurs at the early reprogramming stage, with cells undergoing mesenchymal-to-epithelial transition (MET) for iPSC colony formation (Hussein et al., 2014; Li et al., 2010; Samavarchi-Tehrani et al., 2010). This stage is followed by the second wave that occurs during maturation and stabilization, when the pluripotency regulatory network is activated and stabilized in reprogrammed cells (Buganim et al., 2012; Golipour et al., 2012; Hussein et al., 2014; Polo et al., 2012; Samavarchi-Tehrani et al., 2010). In human cells, the early-to-middle reprogramming stages are characterized by multiple waves of lineage-related gene activation in the order of developmental reversal, with MET occurring at the middle-to-late-reprogramming stage along with pluripotent network activation (Cacchiarelli et al., 2015). This transcriptional alteration in reprogramming is accompanied by epigenomic modifications that suppress somatic gene expression/reactivation and maintain the active pluripotency regulatory network (Cacchiarelli et al., 2015; Hussein et al., 2014; Xu et al., 2016). However, the exact molecular mechanism that ensures successful human cell reprogramming is still poorly defined.

Thus far, induced pluripotency in humans remains a very inefficient and lengthy process. The reprogramming efficiency for human iPSC generation is generally at the low end of the reported range (0.00002–∼1%) in different laboratories, and it usually takes between 3 and 5 weeks for the induced iPSC colonies to appear (Malik and Rao, 2013; Rao and Malik, 2012). Additional reprogramming factors have been reported to enhance the reprogramming efficiency induced by OSKM (Hanna et al., 2009; Maekawa et al., 2011; Silva et al., 2009; Tanabe et al., 2013; Worringer et al., 2014; Yu et al., 2007b; Zhang et al., 2016). *NANOG* is a key gene required for pluripotency maintenance (Pan and Thomson, 2007) and is thought to stabilize reprogramming at the late iPSC induction stage (Hanna et al., 2009; Silva et al., 2009; Yu et al., 2007b). *GLIS1* promotes human iPSC generation and activates *Foxa2* in mouse cell reprogramming to promote MET and to reinforce the activity of the core pluripotent gene network (Maekawa et al., 2011). *LIN28* is exclusively expressed in completely but not partially reprogrammed human iPSCs (Zhang et al., 2016) and promotes the maturation of reprogrammed cells, a major roadblock for successful human iPSC generation (Tanabe et al., 2013). The best known function of *LIN28* is to inhibit Let-7 miRNA maturation to promote the expression of *HMGA2*, *KRAS*, *MYC* (Viswanathan et al., 2009) and HRAS in cancer cells (Cai et al., 2013; Yu et al., 2007a). However, unlike the ectopic expression of *MYC* (Takahashi et al., 2007; Takahashi and Yamanaka, 2006), ectopically expressed *HMGA2*, KRAS or *HRAS* failed to improve human iPSC generation (Worringer et al., 2014). Thus, the exact mechanisms by which these reprogramming factors regulate human cell reprogramming remain elusive.

The canonical WNT/β-CATENIN pathway signals through the T cell factor (TCF)/lymphoid enhancer factor and exerts pleiotropic effects on pluripotency establishment and maintenance. WNT maintains naïve-pluripotent mouse ESCs by suppressing the negative effector *TCF7L1* (formally known as *TCF3*), and stimulating WNT/β-CATENIN activity facilitates mouse iPSC induction (Lluis et al., 2011; Zhang et al., 2014). However, WNT also inhibits mouse ESC proliferation via the effectors *TCF7* and *TCF7L2* (formally known as *TCF1* and *TCF4*, respectively) (Cole et al., 2008; De Jaime-Soguero et al., 2017; Martello et al., 2012). In humans, WNT/β-CATENIN activity is needed for the self-renewal of primed-state human ESCs (Fernandez et al., 2014) or the generation of human iPSCs (Cevallos et al., 2018; Ross et al., 2014). However, enhancing WNT/β-CATENIN and TCF7 signaling promotes differentiation of ESCs or the reprogrammed cells (Cevallos et al., 2018; Davidson et al., 2012; Dravid et al., 2005; Jiang et al., 2013). In addition, the WNT negative regulator *TCF7L1* is needed for the generation of human ESC-like, primed-state pluripotent mouse cells (Hoffman et al., 2013) and maintains human ESC pluripotency by inhibiting primitive streak commitment (Sierra et al., 2018). Thus, WNT activity needs to be carefully controlled in reprogramming. However, how different reprogramming factors regulate canonical WNT signaling for successful reprogramming remains unclear.

In the current study, we used primary human mesenchymal stem cells (MSCs) with very low efficiency in OSKM-mediated reprogramming to study the iPSC induction mediated by OSKM and the reprogramming factors *GLIS1*, *NANOG* and *LIN28* (GNL). We used TRA-1-60, one of the best markers for primed-state pluripotency (Andrews et al., 1984; Chan et al., 2009) and successful iPSC generation (Onder et al., 2012; Tanabe et al., 2013), to monitor the reprogramming process. We found that *NANOG* and *LIN28* (NL), but not *GLIS1*, synergize to stimulate the expression of the Let-7 target *LIN41* and to enhance canonical WNT activity for human iPSC generation. The synergistic effects can be re-enforced by the inhibition of the histone 3 lysine 79 (H3K79) methyltransferase DOT1L, resulting in a more than ∼127-fold increase in TRA-1-60 positive (+) iPSC colonies. Furthermore, we discovered that although the elevated canonical WNT activity facilitates initial reprogramming kinetics, the inhibition of WNT signaling at the middle-to-late-reprogramming stage dramatically enhances the maturation of reprogrammed cells.

## RESULTS

### NL is more efficient than GNL in reprogramming

**Fig. 1.**
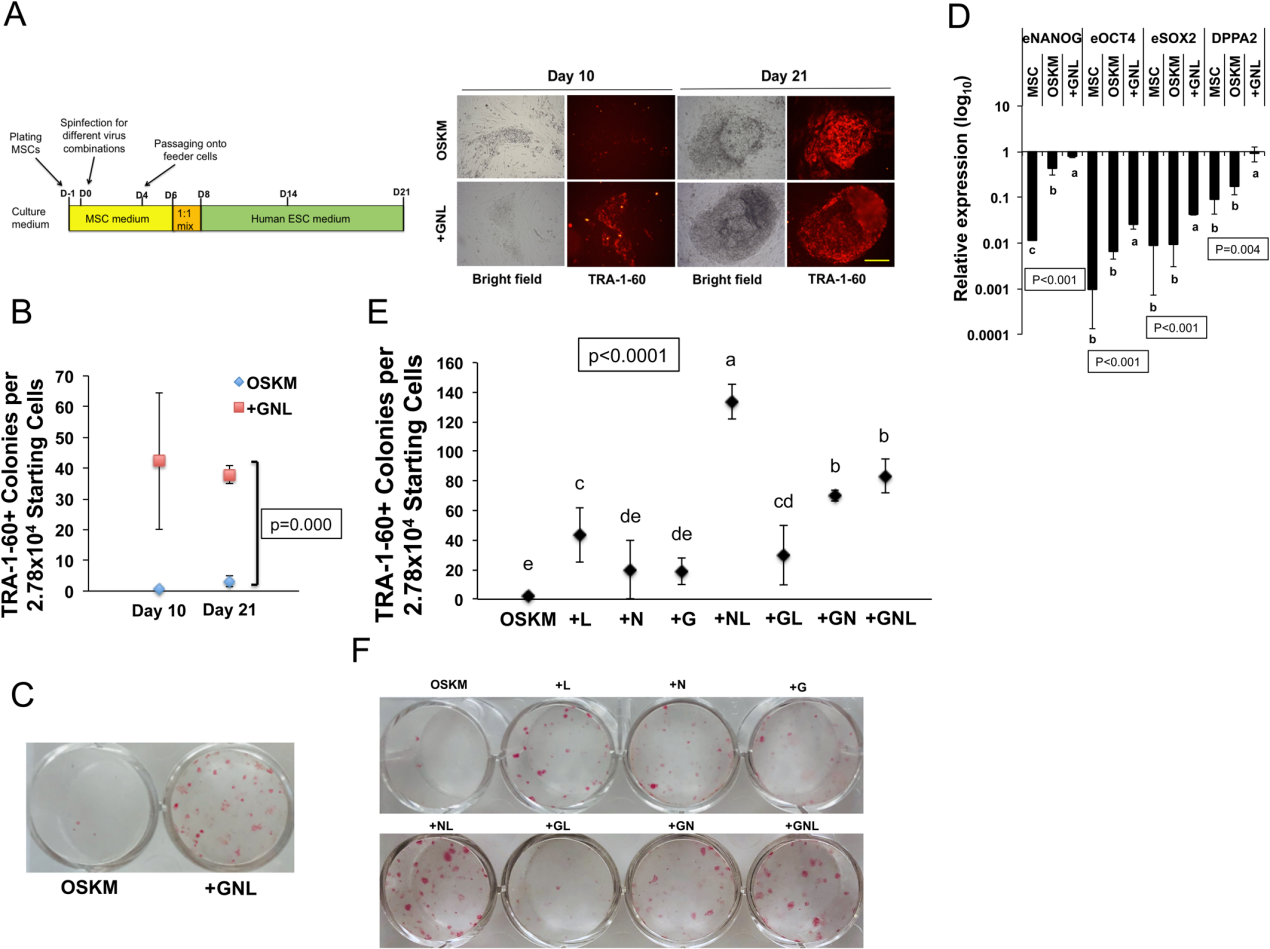
**Effects of *LIN28*, *NANOG* and *GLIS1* on promoting the reprogramming of human MSCs.** (A, left) Schematic diagram of the timeline of human MSC reprogramming. (Right) Representative images of TRA-1-60 immunofluorescence in OSKM- and +GNL-induced colonies of human MSCs on days 10 and 21. Scale bar: 250 μm. (B) Number of TRA-1-60+ colonies in OSKM- and +GNL-mediated reprogramming conditions on days 10 and 21. Scatter plots represent the mean±s.d., *n*=3. (C) Representative images of putative iPSC colonies in OSKM- and +GNL-mediated reprogramming stained with AP on day 21. (D) qRT-PCR results of the pluripotent gene expression in parental MSCs and cells transduced with OSKM or +GNL on reprogramming day 14 relative to the pluripotent gene expression in H9-ESCs. e, endogenous genes. Bars represent the mean±s.d., n=3. (E) Number of TRA-1-60+ colonies in different reprogramming conditions on days 12 and 18. +G, +N and +L represent the addition of *GLIS1*, *NANOG* and *LIN28*, respectively, to OSKM for reprogramming; +NL, +GL, +GN and +GNL represent the respective combinations added to OSKM for reprogramming. Scatter plots represent the mean±s.d., *n*=3. (F) Representative images of putative iPSC colonies under different reprogramming conditions stained with AP on day 21. In all graphs, conditions with different letters are significantly different.

We first hypothesized that coexpressing *GLIS1*, *NANOG* and *LIN28* would greatly enhance OSKM-mediated reprogramming based on their reported individual effects (Hanna et al., 2009; Lee et al., 2017; Maekawa et al., 2011; Yu et al., 2007b; Zhang et al., 2016). Primary MSCs were transduced with OSKM or OSKM+GNL expressed in a retroviral pMXs-vector (Fig. 1A). In the OSKM reprogramming condition, few TRA-1-60+ cell aggregates were observed on day 10 of viral infection and the development of TRA-1-60+ colonies appeared 1–2 weeks later (Fig. 1A,B). In contrast, many ESC-like TRA-1-60+ colonies readily appeared in the GNL condition on day 10 (Fig. 1A,B). The difference in the number of TRA-1-60+ colonies was also correlated with the alkaline phosphatase (AP)-staining of reprogrammed cells (Fig. 1C). Quantitative-reverse transcription PCR (qRT-PCR) analysis on reprogrammed cells at day 14 showed that the GNL combination significantly stimulated the expression of the endogenous (e) pluripotent genes *OCT4*, *SOX2*, *NANOG* and *DPPA2* (Fig. 1D).

We then asked which factor(s) in *GLIS1*, *NANOG* and *LIN28* most effectively promoted reprogramming. We applied the factors individually or in two-factor combinations to the OSKM condition. On day 12, TRA-1-60+ colonies were evident in all other conditions except for the OSKM alone (Fig. S1). The applications of *GLIS1*, *NANOG* or *LIN28* each improved the reprogramming efficiency of human MSCs compared with OSKM, albeit with less efficiency than the GNL combined (Fig. 1E,F). Furthermore, while the *GLIS1* and *NANOG* (GN) combination produced similar reprogramming efficiency to GNL, NL together increased TRA-1-60+ colonies by ∼1.6-fold over that of GNL (Fig. 1E). This result was also correlated with an increase in AP-stained colonies (Fig. 1F). No synergistic effect was observed for the *GLIS1* and *LIN28* (GL) combination (Fig. 1E,F). Thus, among the three additional reprogramming factors, the NL combination most dramatically enhanced OSKM-mediated reprogramming and shortened reprogramming latency by more than 1 week compared with the OSKM condition.

### NL co-stimulate *LIN41* to promote cell epithelialization in reprogramming

**Fig. 2.**
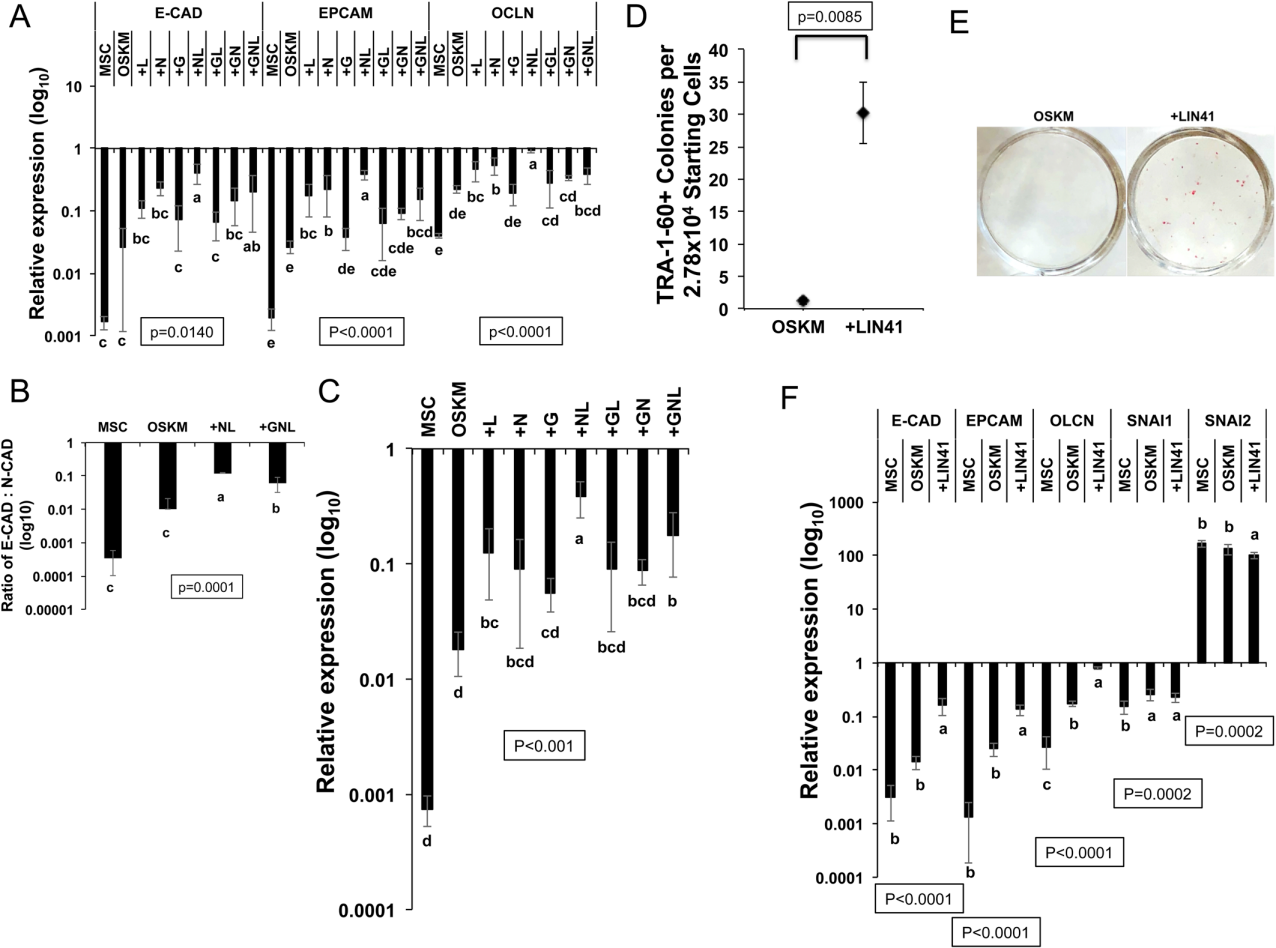
**NL synergize to activate *LIN41* and promote the reprogramming of human MSCs.** (A) qRT-PCR results of epithelial gene expression in MSCs and reprogrammed cells on day 14 under different conditions relative to the epithelial gene expression in H9-ESCs. Bars represent the mean±s.d., *n*=3. (B) Ratio of E-CAD:N-CAD mRNA expression in MSCs and reprogrammed cells on day 14 under different conditions relative to the expression in H9-ESCs. Bars represent the mean±s.d., *n*=3. (C) qRT-PCR results of *LIN41* expression in MSCs and reprogrammed cells on day 14 under different conditions relative to H9-ESC expression. Bars represent the mean±s.d., *n*=3. (D) Number of TRA-1-60+ colonies under the OSKM- and OSKM+*LIN41* (+*LIN41*)-mediated reprogramming conditions on day 12. Scatter plots represent the mean±s.d., *n*=3. (E) Representative images of putative iPSC colonies in OSKM- and +*LIN41*-mediated reprogramming stained with AP on day 18. (F) qRT-PCR results of mesenchymal and epithelial marker expression in parental MSCs and human MSCs reprogrammed with OSKM or +*LIN41* on day 14 relative to H9-ESC expression. Bars represent the mean±s.d., *n*=3. In all graphs, conditions with different letters are significantly different.

To identify a possible mechanism for the NL-enhanced reprogramming, we evaluated the gene expression in reprogrammed cells on day 14. Compared with the OSKM condition, both the addition of NL and GNL significantly improved the expression of core pluripotent genes, including endogenous *NANOG*, *OCT4* and *SOX2*, with no significant difference between the two conditions (Fig. S2). We then asked if NL and GNL differentially regulate the MET process in reprogramming. Compared with OSKM alone or with *GLIS1*, *NANOG* or *LIN28,* NL but not GNL significantly increased the expression of the epithelial markers *E-CADHERIN* (*E-CAD*), *EPCAM* and *OCLN* (Fig. 2A). Moreover, GNL resulted in more decreased *EPCAM* and *OCLN* expression than NL (Fig. 2A). In addition, the ratio of the epithelial marker *E-CAD* versus the mesenchymal marker *N-CAD* (Nakajima et al., 2004; Wang et al., 2016) was increased more significantly in NL than in GNL compared with the OSKM-alone condition (Fig. 2B). No obvious difference was observed in the expression of mesenchymal markers among different reprogramming conditions (Fig. S3). These data indicate that NL synergize to promote cellular epithelialization in reprogramming, while the addition of GLIS1 reduces this synergy.

The mRNA of the ubiquitin ligase LIN41 is targeted by Let-7 miRNAs in *Caenorhabditis elegans*, mice and humans (Ecsedi et al., 2015; Nguyen et al., 2017; Slack et al., 2000; Worringer et al., 2014), and LIN41 plays an important role in overcoming the Let-7 barrier for OSKM-mediated reprogramming from fibroblasts (Worringer et al., 2014). However, although the RNA-binding protein LIN28 directly inhibits the maturation of Let-7 miRNAs (Viswanathan et al., 2008), whether it regulates *LIN41* expression to promote successful reprogramming is not known. We asked if *LIN41* is a downstream target of *LIN28* in reprogramming. Compared with the OSKM condition, the addition of *LIN28* significantly stimulated *LIN41* expression, and this stimulatory effect was synergistically enhanced by NL but not by GL or GN (Fig. 2C). The addition of GNL also exhibited less *LIN41* stimulation than NL (Fig. 2C). Thus, NL co-stimulate the expression of *LIN41* in reprogramming whereas *GLIS1* reduces this effect. We also questioned whether *LIN41* overexpression could improve the OSKM-mediated reprogramming from human MSCs as previously reported from fibroblasts (Worringer et al., 2014). Similar to *LIN28* overexpression (Fig. 1E), ectopic *LIN41* significantly improved OSKM-mediated reprogramming efficiency (Fig. 2D,E; Fig. S4). We further questioned whether *LIN41* regulates MET in reprogramming. Indeed, the overexpression of *LIN41* significantly stimulated the expression of the epithelial markers *E-CAD*, *EPCAM* and *OCLN* (Fig. 2F). Additionally, *LIN41* did not affect the expression of the mesenchymal marker *SNAI1* and only slightly reduced (<25%) the expression of *SNAI2* (Fig. 2F). Thus, our data indicate that *LIN41* functions as a downstream target and effector of *LIN28* and is co-stimulated by NL to promote reprogramming, at least partially by enhancing cellular epithelialization.

### Canonical WNT signaling is synergistically stimulated by NL in reprogramming

**Fig. 3.**
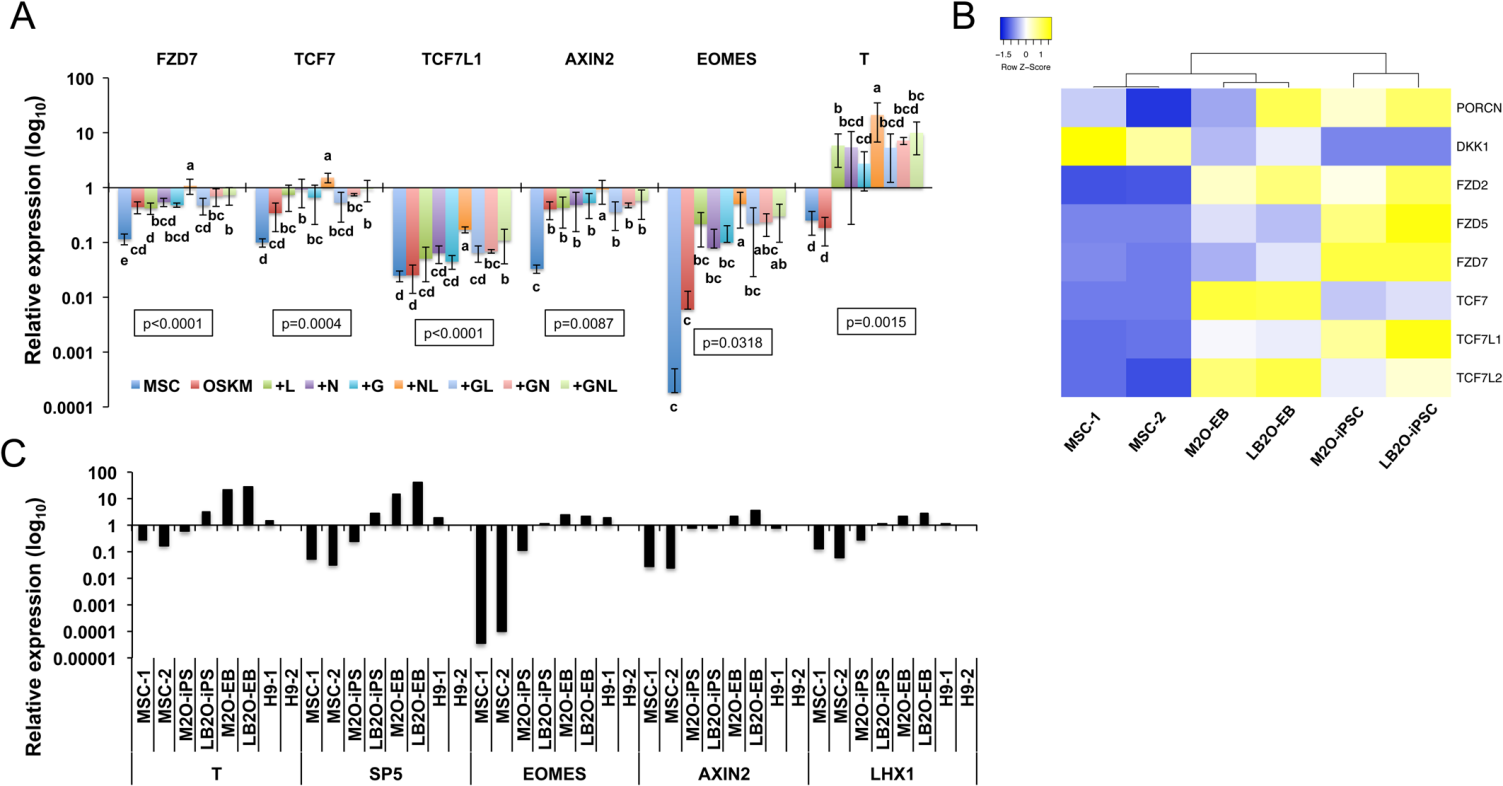
**Activation of canonical WNT signaling in reprogrammed cells and pluripotent stem cells.** (A) qRT-PCR results of the expression of WNT/β-CATENIN pathway components and target genes in MSCs and reprogrammed cells on day 14 under different conditions relative to H9-ESC expression. Bars represent the mean±s.d., *n*=3. Conditions with different letters are significantly different. (B) Heatmap showing the expression of canonical WNT/β-CATENIN pathway regulatory genes in MSCs, two iPSC lines (M_2_O and LB_2_O) and the day-5 EBs differentiated from these iPSCs. (C) qRT-PCR results of the expression levels of WNT/β-CATENIN target genes in MSCs, two iPSC lines (M_2_O and LB_2_O), the day-5 EBs differentiated from these iPSCs and H9-ESCs.

The overexpression of the canonical WNT/β-CATENIN signaling effector *TCF7* initially promoted reprogramming but induced differentiation at late-reprogramming stage (Cevallos et al., 2018). We asked whether WNT activity is modulated by GLIS1, NANOG or LIN28 in reprogramming. Among all conditions, NL substantially stimulated the expression of *FZD7*, the most abundant WNT receptor specific to human ESCs and necessary for pluripotency maintenance (Fernandez et al., 2014). In addition, *TCF7* and the canonical WNT signaling targets *AXIN2*, *EOMES* and *T* (Huggins et al., 2017; Yan et al., 2001) were also greatly activated by NL (Fig. 3A). GNL exerted a smaller stimulatory effect on WNT activity than NL (Fig. 3A). These findings indicate that NL synergistically stimulates canonical WNT activity in reprogramming, while GLIS1 mitigates this stimulatory effect. Meanwhile, NL also moderately but significantly promoted the expression of *TCF7L1* (Fig. 3A), the WNT antagonist and pluripotent marker necessary to prevent hyperactive WNT signaling-induced primitive streak differentiation in human ESCs/iPSCs (Cevallos et al., 2018; Sierra et al., 2018).

We also evaluated whether WNT signaling is elevated in human iPSCs by comparing two previously reported human iPSC lines (Wang et al., 2017) with their parental MSCs and the day 5 embryoid bodies (EBs) differentiated from these iPSCs. Although no obvious difference in the expression of eight canonical WNT ligands (Staal et al., 2008) was found between iPSCs and MSCs (Fig. S5), increased expression of *PORCN*, a membrane bound O-acetyltransferase necessary for WNT ligand secretion (Barrott et al., 2011; Biechele et al., 2011; Proffitt and Virshup, 2012), and decreased expression of *DKK1*, an inhibitor of canonical WNT signaling (Cruciat and Niehrs, 2013) were evident in human iPSCs compared with human MSCs (Fig. 3B). Furthermore, the three WNT receptors reported to enrich in human ESCs – *FZD2*/*5*/*7* (Fernandez et al., 2014), and the WNT effectors *TCF7* and *TCF7L2* – were all increased in iPSCs compared with MSCs (Fig. 3B). We further found that the primitive streak/mesoendoderm markers targeted by canonical WNT signaling, including *T*, *SP5*, *EOMES*, *AXIN2* and *LHX1* (Huggins et al., 2017; Yan et al., 2001), were all highly or moderately upregulated in human iPSCs and ESCs compared with MSCs (Fig. 3C). Taken together, these results indicate that canonical WNT signaling is more active in human pluripotent stem cells than in MSCs and is synergistically stimulated by NL in reprogramming. Additionally, consistent with the known differentiation-stimulating function of fully activated WNT signaling (Sierra et al., 2018), we noticed that compared with iPSCs and MSCs, EBs exhibited markedly elevated WNT ligands (Fig. S5) and WNT effectors *TCF7*/*TCF7L2* only moderately increased WNT antagonist *TCF7L1* (Fig. 3B).

### Inhibiting H3K79 methyltransferase enhances NL-stimulated reprogramming, while blocking WNT signaling promotes iPSC maturation

**Fig. 4.**
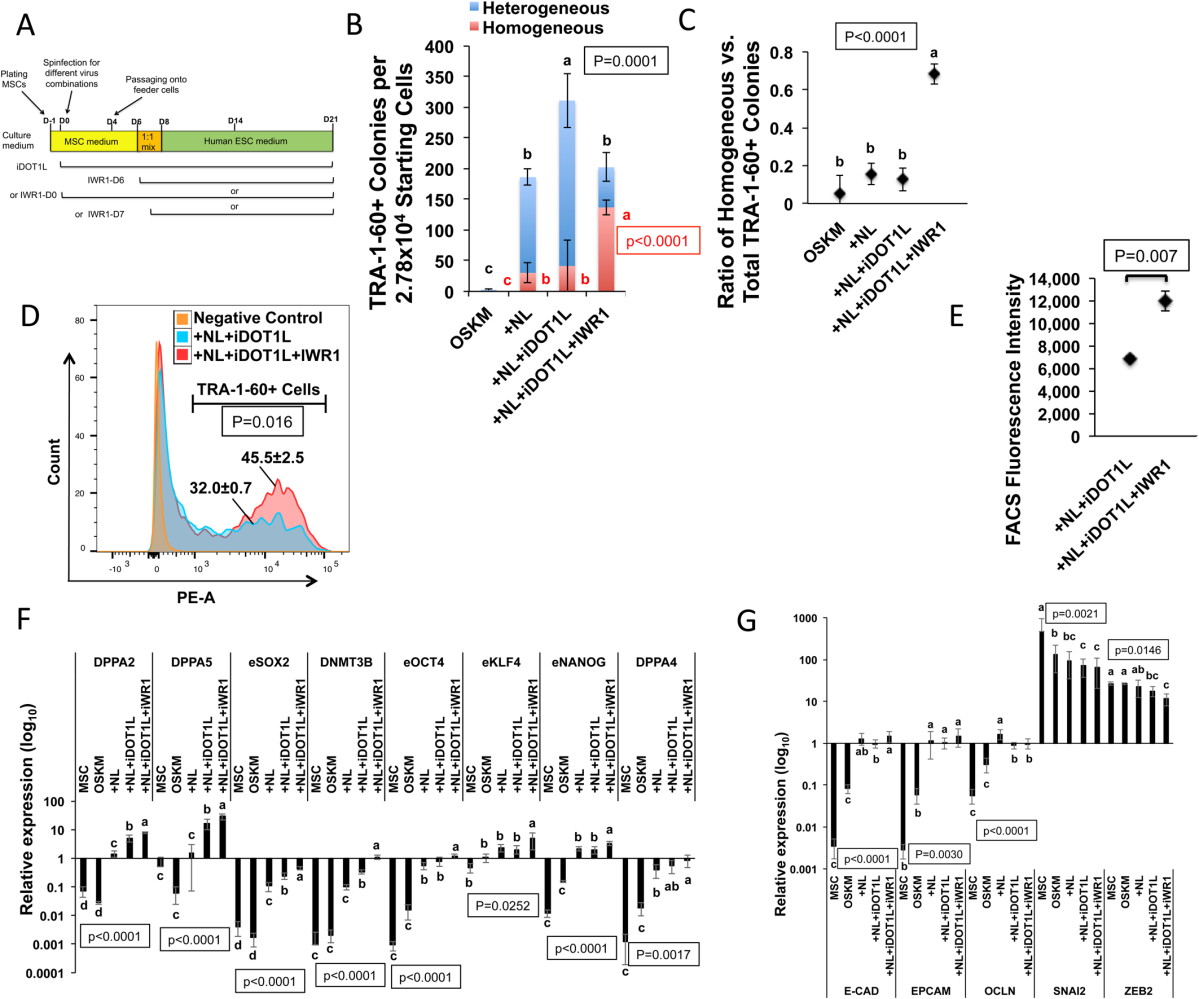
**Effects of inhibiting H3K79 methylation and the WNT signaling pathway on reprogramming.** (A) Schematic diagram showing the timeline of iDOT1L and IWR1 administration in reprogramming. (B) Numbers of homogeneous (red), heterogeneous (blue) and total (red plus blue) TRA-1-60+ colonies in the OSKM condition and the other reprogramming conditions on day 12. +NL, +NL+iDOT1L and +NL+iDOT1L+IWR1 represent the addition of NL alone or NL plus inhibitor(s) to OSKM for reprogramming. Bars represent the mean±s.d., *n*=3. Letters and *P*-values shown in red and black represent the statistics for the numbers of homogeneous and total TRA-1-60+ colonies, respectively. (C) Ratio of homogeneous versus total TRA-1-60+ colonies in the OSKM condition and the other reprogramming conditions as indicated in B on day 12. Scatter plots represent the mean±s.d., *n*=3. (D) FACS analysis of cellular TRA-1-60 immunofluorescence on reprogramming day 14 with or without WNT inhibition. The percentage of TRA-1-60+ cells out of the total reprogrammed cells is shown as the mean±s.d., *n*=3. (E) Median fluorescence intensity of TRA-1-60+ cells as determined by FACS analysis on reprogramming day 14 with or without WNT inhibition. Scatter plots represent the mean±s.d., *n*=3. (F) qRT-PCR results of pluripotent marker gene expression in MSCs and reprogrammed cells on day 14 under the conditions described in B relative to H9-ESC expression. Bars represent the mean±s.d., *n*=3. (G) qRT-PCR results of epithelial and mesenchymal gene expression in MSCs and reprogrammed cells on day 14 under the conditions described in B relative to H9-ESC expression. Bars represent the mean±s.d., *n*=3. In all graphs, conditions with different letters are significantly different.

H3K79 dimethylation (H3K79me2) is a barrier of reprogramming from human fibroblasts (Onder et al., 2012). We asked if inhibiting H3K79me2 would further enhance the NL-mediated improvement in reprogramming efficiency. An inhibitor of the H3K79 methyltransferase DOT1L (iDOT1L) (Onder et al., 2012) was added at day 0 of reprogramming (Fig. 4A). The addition of iDOT1L enhanced OSKM-mediated reprogramming (Figs S6 and S7). Similarly, iDOT1L also enhanced the reprogramming mediated by OSKM plus a polycistronic NL expression (used hereafter in all +NL conditions), resulting in an ∼127-fold increase in total TRA-1-60+ colonies compared with the OSKM condition, in contrast to the ∼76-fold increase in the NL condition with no iDOT1L (Fig. 4B).

As we found that NL stimulate canonical WNT signaling in reprogramming, and hyperactive WNT causes human iPSC/ESC differentiation (Cevallos et al., 2018; Sierra et al., 2018), we wondered if inhibiting WNT would improve the NL-enhanced reprogramming to a greater extent. A canonical WNT inhibitor IWR1 (Chen et al., 2009) has been shown to improve the maintenance of human ESC self-renewal (Kim et al., 2013). We added IWR-1 at day 6 of reprogramming, when iPSC colony formation was evident (Fig. 4A). Interestingly, the addition of IWR1 produced flat-shaped iPSC colonies that more morphologically resemble human ESCs than the other conditions on day 12 (Fig. S8). Furthermore, the colonies observed with the addition of IWR1 exhibited brighter and more homogeneous TRA-1-60 fluorescence (Fig. S9). We therefore counted both the homogeneous and heterogeneous TRA-1-60+ colonies in reprogramming. Although the number of total (homogenous and heterogeneous) TRA-1-60+ colonies was greatest in the NL+iDOT1L condition (Fig. 4B), the ratio of homogeneous versus total TRA-1-60+ colonies remained low (<20%) (Fig. 4C). However, compared with the NL+iDOT1L condition, the NL+iDOT1L+IWR1 reprogramming condition exhibited striking increase in the number and ratio of homogeneous TRA-1-60+ colonies (Fig. 4B,C). The positive effects of iDOT1L and IWR1 on NL-enhanced TRA-1-60+ colony formation were also correlated with the AP-staining of induced colonies at reprogramming day 18 (Fig. S10). The fluorescence-activated cell sorting (FACS) further confirmed that compared with the NL+iDOT1L condition, the NL+iDOT1L+IWR1 condition significantly increased the percentage of TRA-1-60+ cells on day 14 (Fig. 4D); the median fluorescence intensity of TRA-1-60+ cells was also increased ∼1.8-fold in the NL+iDOT1L+IWR1 condition (Fig. 4E).

Consistent with the increase in total TRA-1-60+ colonies, compared with the NL condition, NL+iDOT1L increased the expression of the pluripotency markers *DPPA2*/*5* (Qian et al., 2016; Tung et al., 2013) and some late-reprogramming stage markers, including endogenous *SOX2* and *DNMT3B* (Buganim et al., 2012; Cacchiarelli et al., 2015; Takahashi et al., 2014) (Fig. 4F). However, compared with the NL and NL+iDOT1L conditions, the addition of IWR1 not only further enhanced the expression of these genes mentioned above, but also increased the expression of more core pluripotency markers, including endogenous *OCT4*, *NANOG*, *KLF4* and *DPPA4* (Cacchiarelli et al., 2015) (Fig. 4F). This was correlated with the increased TRA-1-60+ cell population as well as the enhanced TRA-1-60 fluorescence intensity in the NL+iDOT1L+IWR1 condition, and indicates a reinforcement of pluripotency network activity for the NL+iDOT1L enhanced reprogramming by inhibiting WNT.

We asked whether the addition of iDOT1L or IWR1 would impact MET in reprogramming. While NL stimulated dramatic epithelial marker expression compared with OSKM, it exhibited no obvious effect on mesenchymal markers similarly as we had observed (Fig. 4G; Fig. S3). However, compared with the OSKM condition, the NL+iDOT1L condition significantly decreased the expression of the mesenchymal markers *SNAI2* (∼46%) and *ZEB2* (∼33%), and the addition of IWR1 further reduced *ZEB2* expression (∼57%) (Fig. 4G). These data indicate that NL are the main driving forces underlying cell epithelialization in reprogramming. Additionally, iDOT1L could enhance reprogramming by suppressing the expression of mesenchymal markers, which can be further enhanced by the addition of IWR1. All these underpin the activation of pluripotency network and promote the maturation of reprogrammed cells.

To verify the pluripotency of putative iPSCs, we picked the homogeneous TRA-1-60+ colonies on reprogramming days 18–21 from different conditions (NL, NL+iDOT1L, and NL+iDOT1L+IWR1). These cells readily expanded in a mTeSR1 feeder-free condition (Ludwig et al., 2006a,b). iDOT1L and IWR1 were removed during the expansion. These iPSC lines exhibited silencing of all transgenes at passage 11 (Fig. S11) and expressed pluripotent genes/proteins at similar levels as human ESCs (Figs S11 and S12). To confirm their differentiation capacity, iPSCs established from different conditions were subjected to EB differentiation (Fig. S13). qRT-PCR and immunostaining analyses of EBs at day 5 demonstrated significant activation of lineage markers for three germ layers (Figs S14 and S15).

### iDOT1L treatment enhances NL-stimulated WNT and LIN41 activities, and LIN41 expression contributes to WNT activation in reprogramming

**Fig. 5.**
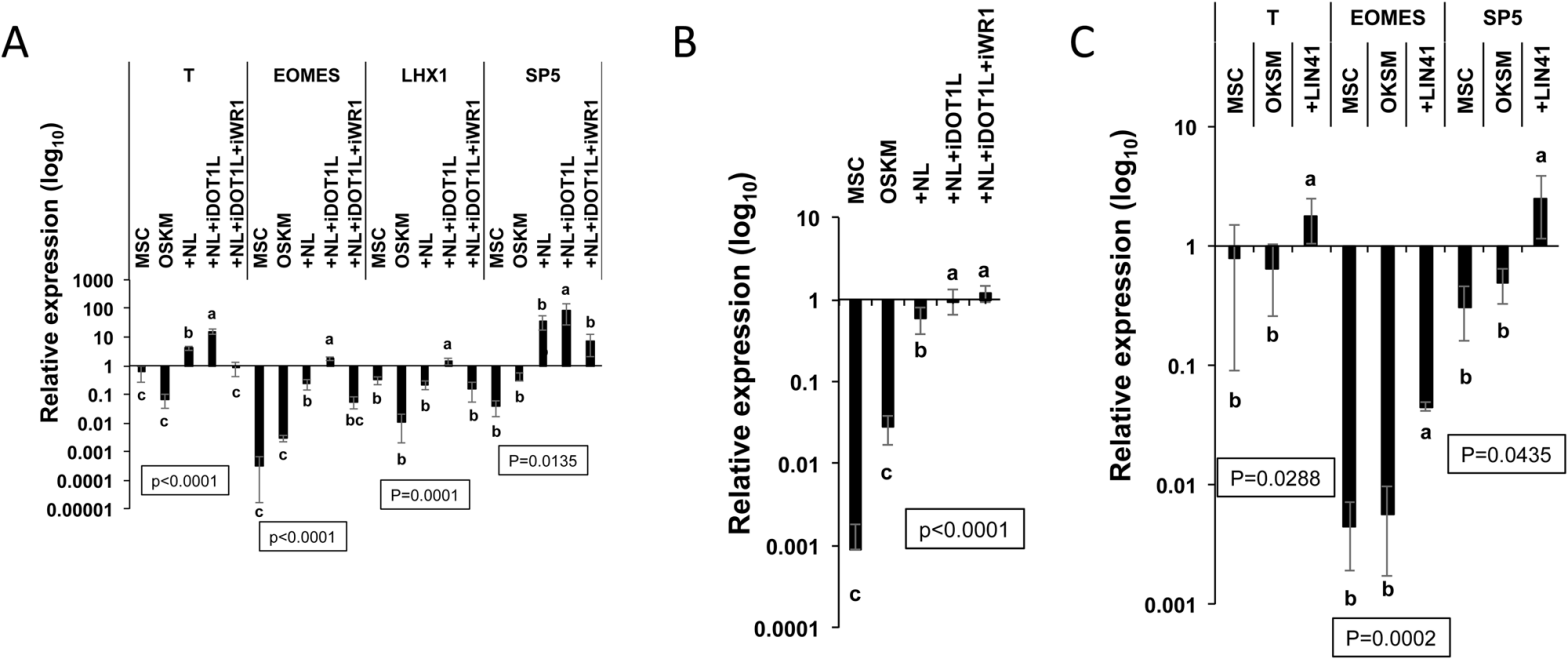
**iDOT1L treatment enhances WNT and LIN41 activities in reprogramming.** (A) qRT-PCR results showing the expression of WNT/β-CATENIN target genes in MSCs and reprogrammed cells by OSKM, +NL, +NL+iDOT1L and +NL+iDOT1L+IWR1 on day 14 under the conditions described in Fig. 4B relative to H9-ESC expression. Bars represent the mean±s.d., *n*=3. (B) qRT-PCR results of NL- and iDOT1L-induced LIN41 expression on reprogramming day 14 under the conditions described in A relative to H9-ESC expression. Bars represent the mean±s.d., *n*=3. (C) qRT-PCR results of the OSKM- and OSKM+*LIN41* (+*LIN41*)-mediated reprogramming conditions WNT target genes on reprogramming day 14 relative to H9-ESC expression. Bars represent the mean±s.d., *n*=3. In all graphs, conditions with different letters are significantly different.

We asked if inhibiting H3K79me2 by iDOT1L would affect the NL-stimulated WNT activity. Interestingly, we found that iDOT1L treatment further enhanced the expression of WNT target genes induced by NL in reprogramming (Fig. 5A). As expected, IWR1 inhibited the WNT activity co-stimulated by NL and iDOT1L (Fig. 5A).

We have shown that NL synergistically stimulate *LIN41* expression and that *LIN41* enhances the OSKM-mediated reprogramming of MSCs (Fig. 2C–E). We further questioned if *LIN41* expression is regulated by iDOT1L treatment and WNT inhibition. Compared with the NL condition, the addition of iDOT1L further enhanced *LIN41* expression in reprogramming (Fig. 5B). IWR1, however, did not significantly alter *LIN41* expression level (Fig. 5B). We also asked whether *LIN41* could regulate WNT activity by analyzing the reprogrammed cells in OSKM and OSKM+*LIN41* conditions (Fig. 2D,E). Compared with the OSKM condition, the OSKM+*LIN41* condition exhibited significantly enhanced expression of canonical WNT targets, including *T*, *EOMES* and *SP5* (Fig. 5C). These results indicate that NL and iDOT1L co-stimulate LIN41 expression, which is independent of WNT signaling, and LIN41 participates in the activation of canonical WNT signaling in reprogramming, which is consistent with what we had observed for NL (Fig. 3A).

### The activities of WNT and LIN41 are critical for NL- and iDOT1L-mediated reprogramming

**Fig. 6.**
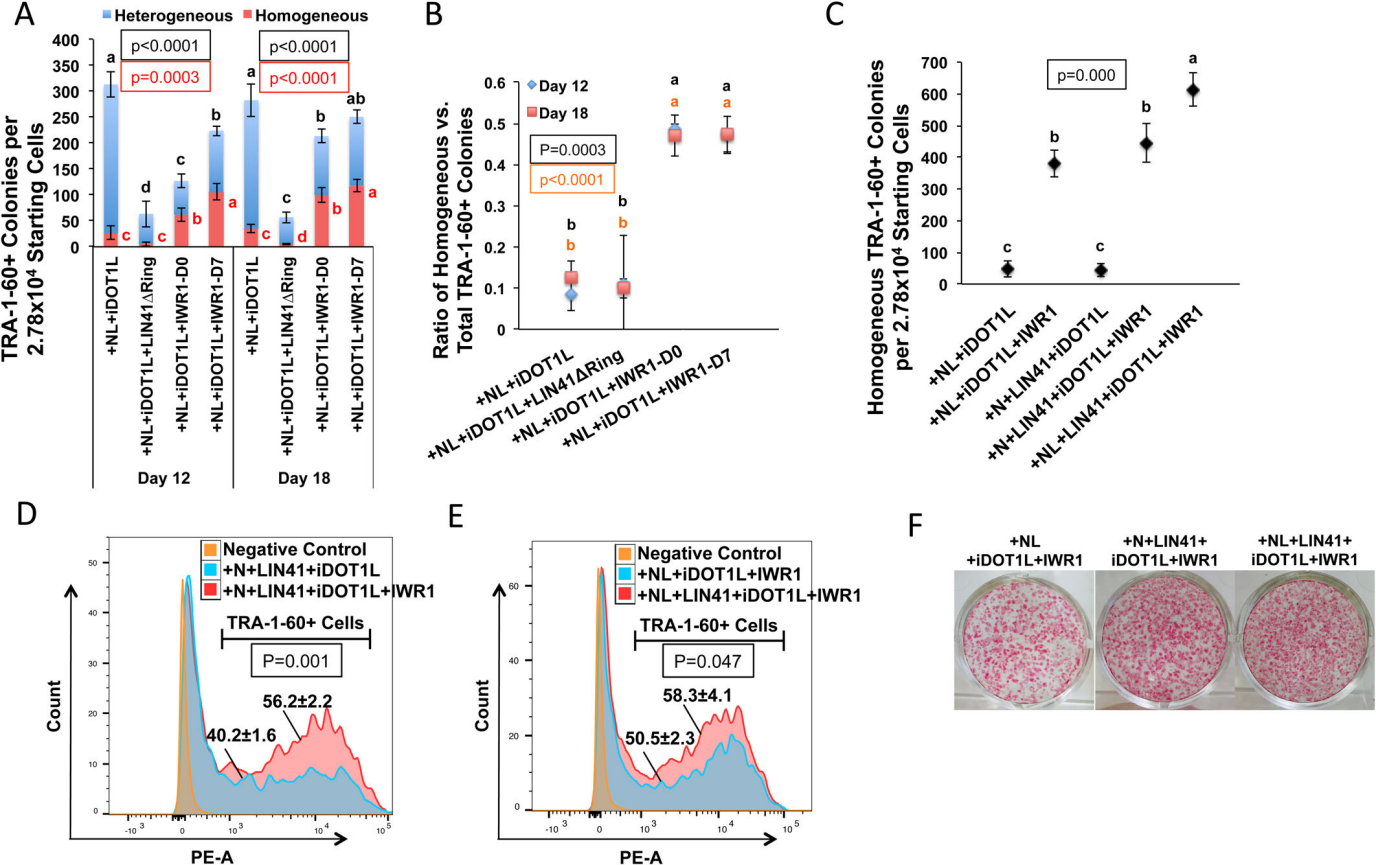
***LIN41* and WNT play critical roles in the enhancement of human cell reprogramming induced by NL and iDOT1L.** (A) Effects of dominant-negative *LIN41* overexpression and the WNT inhibition applied from reprogramming day 0 or day 7 on the numbers of homogeneous (red), heterogeneous (blue) and total (red plus blue) TRA-1-60+ colonies. Bars represent the mean±s.d., *n*=3. Letters and *P*-values shown in red and black represent the statistics for the number of homogeneous and total TRA-1-60+ colonies, respectively. (B) Ratio of homogeneous versus total TRA-1-60+ colonies under the different reprogramming conditions indicated in C. Scatter plots represent mean±s.d., *n*=3. Letters and *P*-values shown in black and red represent the day 12 and day 18 statistics, respectively. (C) Effects of *LIN41* overexpression and IWR1 addition beginning on reprogramming day 6 on the formation of homogeneous TRA-1-60+ colonies on reprogramming day 12. Scatter plots represent the mean±s.d., *n*=3. (D) FACS analysis of cellular TRA-1-60 immunofluorescence on reprogramming day 14 in the +N+*LIN41*+iDOT1L condition with or without WNT inhibition. The percentage of TRA-1-60+ cells out of total reprogrammed cells is shown as the mean±s.d., *n*=3. (E) FACS analysis of cellular TRA-1-60 immunofluorescence on reprogramming day 14 in the +NL+iDOT1L+IWR1 and +NL+*LIN41*+iDOT1L+IWR1 conditions. The percentage of TRA-1-60+ cells out of total reprogrammed cells is shown as the mean±s.d., *n*=3. (F) Representative pictures of putative iPSC colonies stained with AP on reprogramming day 21. In all graphs, conditions with different letters are significantly different.

We wondered how the LIN41 and WNT activities contribute to the enhanced reprogramming by the NL and iDOT1L addition. A dominant-negative LIN41 mutant with an N-term RING domain deletion (pMXs-LIN41ΔRing) (Worringer et al., 2014) was added to the NL+iDOT1L condition. Additionally, IWR1 was added to the NL+iDOT1L condition from initial (day 0) or middle-to-late-reprogramming (day 7) to evaluate the effect of WNT signaling on reprogramming (Fig. 4A). The numbers of homogenous/heterogeneous TRA-1-60+ colonies were counted on reprogramming days 12 and 18 (Fig. 6A). Compared with the NL+iDOT1L condition, LIN41ΔRing reduced the total TRA-1-60+ colonies to only ∼20% of the NL+iDOT1L condition on both days 12 and 18 (Fig. 6A). This result also correlated with the reduced number of AP-stained colonies in the LIN41ΔRing condition on day 18 (Fig. S16). These data demonstrate that LIN41 plays a critical role in NL-induced iPSC colony formation. For WNT inhibition during reprogramming, we found that on day 12, the addition of IWR1 from day 0 reduced the number of total TRA-1-60+ colonies to ∼41% of those in the NL+iDOT1L condition, in contrast to the reduction to ∼71% when IWR1 was added from day 7 (Fig. 6A). However, on day 18, the total number of TRA-1-60+ colonies increased to ∼75% and ∼89% of the NL+iDOT1L condition for IWR1 treatments from day 0 and 7, respectively (Fig. 6A). Additionally, the ratio of homogeneous versus total TRA-1-60+ colonies was similar regardless whether IWR1 was applied from day 0 or 7, and was significantly greater than the NL+iDOT1L condition on days 12 and 18 (Fig. 6B). These data indicate that the activated WNT signaling by NL and iDOT1L plays a significant role in facilitating the kinetics of initial iPSC colony development. However, the subsequent maturation of reprogrammed cells in these colonies requires the inhibition of WNT activity.

**Fig. 7.**
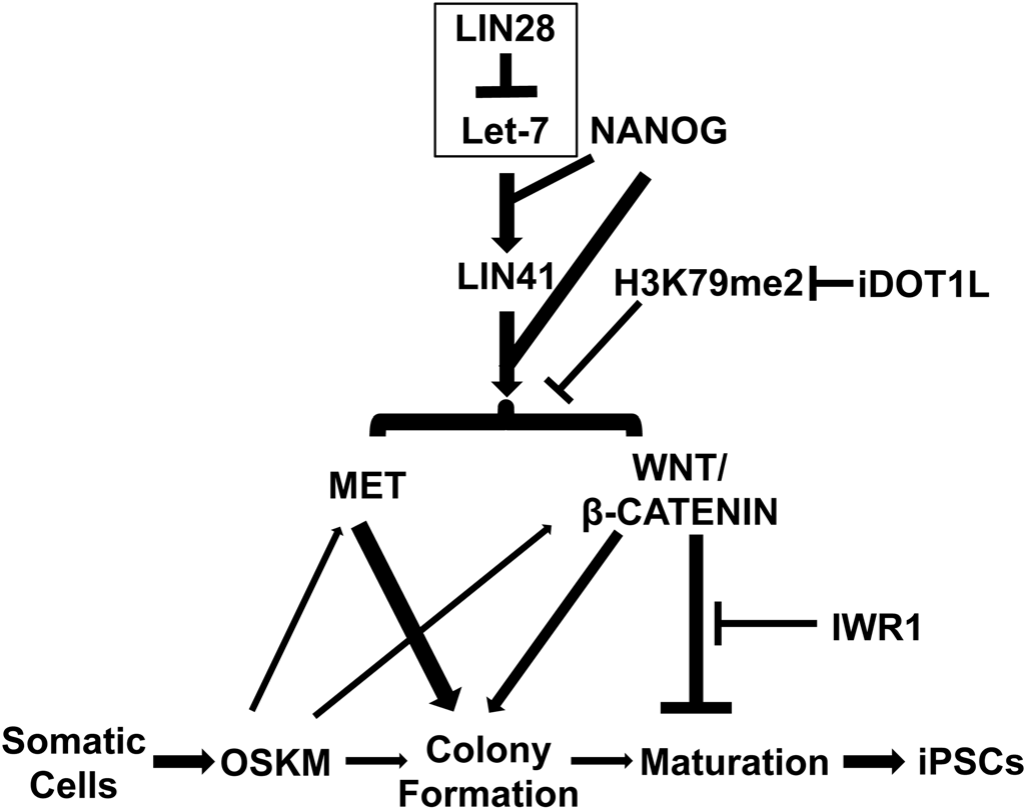
**Schematic model for the enhanced reprogramming of human somatic cells by LIN28, NANOG and iDOT1L.** Proposed model: the enhanced human somatic cell reprogramming by NANOG and LIN28 involves their synergy in activation of LIN41, which is a target of LIN28/Let-7 pathway. LIN41 can replace LIN28 to synergize with NANOG, achieving the same amplification of reprogramming efficiency as the NANOG and LIN28 combination. This synergy can be further enhanced with the inhibition of H3K79 methyltransferase DOT1L. The strong stimulation of MET and activation of the canonical WNT signaling pathway contribute to the massive colony formation in the optimized reprogramming system. For the WNT pathway, despite its positive role in promoting colony formation, hyperactivation of WNT triggers the differentiation of the emerging presumptive colonies. Hence, inhibition of the WNT pathway by IWR1 at late stage of reprogramming can promote the maturation of the emerging colony, without compromising the induced iPSC colony numbers. A thicker line within the graph indicates a stronger stimulation or inhibition compared with a thinner line toward the same biological effect.

As we found that *LIN41* is a critical downstream effector of *LIN28* in reprogramming, we asked whether *LIN41* could replace *LIN28* in synergizing with *NANOG* (N+*LIN41*) for reprogramming. In striking similarity to the NL+iDOT1L condition, when IWR1 was added (from day 6), the replacement of *LIN28* with *LIN41* (N+*LIN41*+iDOT1L) induced a ∼10-fold increase in the homogeneous TRA-1-60+ colonies compared with the condition without IWR1 on day 12 (Fig. 6C). FACS analysis further revealed a significant increase in the TRA-1-60+ cell population and fluorescence intensity when IWR1 was added to the N+*LIN41*+iDOT1L condition (Fig. 6D; Fig. S17). These results indicate that *LIN41* can replace *LIN28* to synergize with *NANOG* in reprogramming. Furthermore, when *LIN41* was coexpressed with NL, the homogeneous TRA-1-60+ colonies further increased by >1.6-fold over the NL+iDOT1L+IWR1 condition (Fig. 6C). The increase in the TRA-1-60+ cell population by NL+LIN41+iDOT1L+IWR1 condition was also confirmed by FACS analysis (Fig. 6E). These results correlated with the number of AP-stained colonies at 3 weeks of reprogramming, showing a dramatic generation of AP+ colonies (Fig. 6F). Thus, the efficiency of establishing homogeneous TRA-1-60+ colonies from the initial MSCs by combined NL and *LIN41* overexpression was ∼2% (Fig. 6C), in contrast to the ∼0.0004% efficiency under the OSKM condition (Fig. 4B), representing a 1000-fold increase in reprogramming efficiency. Taken together, the results in our study demonstrated that NL and iDOT1L promote reprogramming efficiency and kinetics via mechanisms that include *LIN41* stimulation, MET and canonical WNT activation, and that the inhibition of WNT at the middle-to-late-reprogramming stage dramatically facilitates the maturation of reprogrammed cells (Fig. 7).

## DISCUSSION

Human somatic cell reprogramming by OSKM or OSNL remains highly inefficient. This inefficiency is likely due to the required coordination of many cellular events to overcome the reprogramming roadblocks, including the activation of the cell cycle and MET, the silence of lineage gene expression, metabolic resetting, and the complete activation of the pluripotent regulatory network (Brouwer et al., 2016; Xu et al., 2016). We found that among the combinations of *GLIS1*, *NANOG* and *LIN28*, OSKM-mediated reprogramming is synergistically stimulated by the NL combination, while *GLIS1* mitigates this synergy. ‘The pioneering model’ of OSKM-mediated reprogramming showed that OSK factors bind to the shared genomic targets at the initial reprogramming stage to remodel chromatin with the assistance of *MYC*. This gradually enhances the binding of reprogramming factors to the genomic loci and the activation of the endogenous pluripotent network (Soufi et al., 2012). However, how NL factors induce iPSCs or improve reprogramming efficiency is not completely understood (González and Huangfu, 2016). Let-7 miRNAs promote differentiation by inhibiting the genes targeted by the core reprogramming factors *OCT4*, *SOX2* and *NANOG*, and the inhibition of Let-7 increased reprogramming efficiency in mice (Melton et al., 2010) and humans (Worringer et al., 2014). However, whether Lin28 can regulate LIN41 in reprogramming has not been demonstrated. We demonstrated that in reprogramming, *LIN28* significantly stimulates the expression of *LIN41*, the direct target of Let-7, consistent with the demonstrated inhibitory function of LIN28 protein for Let-7 miRNA maturation (Heo et al., 2008; Newman et al., 2008; Rybak et al., 2008; Viswanathan et al., 2009). We also found that NL synergize in *LIN41* activation and that this effect can be further augmented by inhibiting the H3K79 methyltransferase *DOT1L*. Similar to NL, *LIN41* overexpression significantly promoted epithelial gene expression in reprogramming. We further showed that a dominant-negative mutation of *LIN41* greatly suppressed the enhanced reprogramming by NL+iDOT1L. Finally, we showed that the combination of *NANOG* and *LIN41* resulted in similar reprogramming efficiency to NL. Thus, for the first time, our findings established *LIN41* as a key downstream effector of the *LIN28*- and *NANOG*-mediated enhancement in human iPSC generation and indicate that this mechanism occurs, at least partially, by promoting cellular epithelialization.

The effect of the canonical WNT/β-CATENIN pathway on reprogramming remains contradictory. A recent report showed that in OSKM-mediated human cell reprogramming, the hyperactivation of WNT at early stages promoted iPSC colony formation, while it stimulated differentiation at late stages (Cevallos et al., 2018). However, in mouse cell reprogramming, WNT inhibited early-stage reprogramming but promoted late-stage reprogramming (Ho et al., 2013). Both *GLIS1* and *NANOG* have been indicated to activate certain components of the WNT pathway in reprogramming (Maekawa et al., 2011; Marucci et al., 2014), and WNT and *LIN28* co-amplify the expression of their target genes in cancer cells (Tu et al., 2015). However, how canonical WNT activity is regulated by the reprogramming factors for iPSC generation is unclear. We found that NL factors exert synergistic effects in the stimulation of WNT/β-CATENIN activity and that this stimulation can be further enhanced by inhibiting DOT1L. Additionally, we found that NL- and iDOT1L-activated canonical WNT signaling contributes to the kinetics of initial iPSC colony development. We also found that similar to *LIN28*, *LIN41* plays a positive role in stimulating WNT activity in reprogramming. Furthermore, we found that the inhibition of WNT activity from the middle-to-late-reprogramming stage dramatically improved the homogeneity of TRA-1-60+ colonies and the population/intensity of TRA-1-60 expression in reprogrammed cells. This finding correlates with the enhanced expression of late-reprogramming stage markers in reprogrammed cells (Fig. 4G). Our study thus unveiled a mechanism of the synergistic stimulation of *LIN41* and canonical WNT activities by NL and the inhibition of H3K79me2 to ensure highly efficient reprogramming from human primary somatic cells; moreover, the suppression of WNT signaling further improved the maturation of reprogrammed cells (Fig. 7). Exactly how *LIN41* works with *NANOG* to activate MET and WNT activities in reprogramming warrants further investigation. The robust reprogramming system we described here would be of great value to study reprogramming mechanisms using primary cell culture and to rapidly establish the appropriate quality and quantity of mature human iPSCs for differentiation studies as well as for further translational research and applications.

## MATERIALS AND METHODS

### Chemicals and DNA constructs

The DOT1L inhibitor EPZ004777 (iDOT1L) was purchased from AOBIOUS Inc. (Gloucester, MA, USA). WNT inhibitor IWR1 was purchased from Selleckchem (Houston, TX, USA). The constructs pMXs-OCT4, NANOG, LIN28A and GLIS1 were purchased from Addgene (Cambridge, MA, USA). Construction of the polycistronic vector pMXs-KLF4, MYC and SOX2 (KMS) was described in our previous study (Wang et al., 2017). To clone the pMXs-GNL or NL polycistronic vector, the coding sequences for human NANOG, LIN28A and GLIS1 were PCR-amplified from the above-mentioned Addgene constructs. The amplified DNA sequences for each gene were then inserted into linearized pMXs vectors (Cell Biolabs, San Diego, CA, USA) using an In-Fusion kit (Clontech Inc., Mountain View, CA, USA). 2A sequences (Carey et al., 2009; Ryan and Drew, 1994; Ryan et al., 1991) were inserted between each gene.

### Retrovirus packaging with 293T cells

293T cells were plated onto six-well plates at 2.5×10^6^ cells/plate. The next day, pMXs constructs, PUMVC and pCMV-VSVG (Addgene) plasmids were co-transfected into 293T cell using Fugene 6 reagent (Promega, Madison, WI, USA). Cell culture media containing retroviruses were harvested at 48 and 72 h post-transfection and filtered through a 0.8 μm filter. The viruses were stored in −70°C before use.

### Human somatic cell reprogramming

Primary human umbilical cord-derived MSCs from ATCC (Manassas, VA, USA) were used to carry out the reprogramming experiments. MSCs were maintained with low serum mesenchymal stem cell growth kit (ATCC). For reprogramming, on day −1, MSCs at passages 5–6 were plated onto six-well tissue-culture plates at a density of 5×10^5^ cells/plate. On day 0, retroviruses carrying OSKM and other reprogramming factors were added to the cell culture with 10 μg/ml polybrene and spinfected at 650 g for 45 min. The infected cells on day 4 were passaged onto mitomycin C-treated mouse embryonic fibroblast (MEF) feeders in the presence of 10 μM Y-27632 (Selleckchem) ROCK inhibitor. On day 4, the medium was changed to a 1:1 mix of UC-MSCs medium and human ESC medium. Starting from day 6, the cells were maintained in complete human ESC medium, which contains 20% knockout serum replacement (KSR) in DMEM/F12, supplemented with 1× NEAA, 1× Glutamax, 0.5× penicillin and streptomycin, 4 ng/ml human FGF2 (all from Thermo Fisher Scientific, Waltham, MA, USA) and 1× β-mercaptoethanol (Merck Millipore, Billierica, MA, USA). iDOT1L (3.3 μM) and IWR1 (2.5 μM) were added in reprogramming as specified in the main text and maintained thereafter. For iPSC line characterization, TRA-1-60+ colonies were picked on days 18–21 of reprogramming and grown in human ESC medium on MEF feeders. The colonies were dispatched by 1 mg/ml dispase (Thermo Fisher Scientific) at passage 2, transferred to a Matrigel (Corning Inc., NY, USA) feeder-free system and then cultured in mTeSR1 medium (STEMCELL Technologies, Inc., Vancouver, Canada) for expansion.

### EB formation

EB formation experiments were carried out with human iPSC lines at passage 11. When growing to 70–80% confluency with mainly middle-size colonies, the cells were treated with freshly prepared 1 mg/ml dispase for 30 min and removed from the plate by pipetting. After three washes with DMEM/F12, the cells were then plated onto low-adhesive petri dishes in EB formation medium, which is human ESC medium without FGF2. EBs at day 5 were harvested for RNA isolation and gene expression analysis. For immunofluorescence analysis, EBs were treated by TrypLE (Thermo Fisher Scientific) on day 4 and plated onto gelatin-coated plates. The cells were subjected to immunofluorescence staining on day 14.

### Immunofluorescence and TRA-1-60 live staining

Putative iPSC lines at passage 11 were subjected immunofluorescence-staining for pluripotent marker expression. The cells from EB differentiation were studied for lineage differentiation markers. For immunofluorescence, the cells were first fixed in 4% PFA for 15 min at room temperature. Following fixation, the cells were treated with 0.5% Triton X-100 in PBS for 15 min at room temperature for cell membrane permeabilization. After blocking, the cells were incubated in primary antibodies for 2 h at 37°C, followed by secondary antibodies at room temperature for 1 h. Cells were counter-stained with DAPI and imaged under a Nikon fluorescence microscope. Primary antibodies including rabbit anti-OCT4 (Merck Millipore), rabbit anti-SOX2 (Abcam, San Francisco, CA, USA), rabbit anti-NANOG (Merck Millipore), NL-557 conjugated OTX2, NL-493 conjugated GATA4 (R&D Systems, Minneapolis, MN, USA) and mouse anti-SMA (Sigma-Aldrich, St. Louis, MO, USA) were used at 1:100 dilution. Alexa Fluro 488 conjugated goat anti-rabbit or goat anti-mouse secondary antibody (Cell Signaling Technology, Danvers, MA, USA) was used in 1:500 dilution.

For TRA-1-60 live staining, the cells in different reprogramming conditions were stained with GloLIVE TRA-1-60 live-stain antibodies (R&D Systems) according to the manufacturer's protocol. Briefly, the cells were incubated in reprogramming media containing TRA-1-60 antibodies at 1:100 dilution for 30 min. The cells were then washed with DPBS and continued to be cultured in reprogramming media. For colony counting, the stained colonies were visualized under a Nikon fluorescence microscope, with homogenous and heterogeneous TRA-1-60+ colony numbers counted. For FACS analysis, cells were treated with TrypLE and resuspended in reprogramming media. Stained cells were then analyzed with a BD LSRFortessa flow cytometer with fluorescence excitation at 557 nm (BD Biosciences, San Jose, CA, USA). FlowJo software was used for data analysis.

### qRT-PCR analysis

Total RNAs were isolated from parental MSCs, reprogrammed MSCs, or putative iPSCs, or human H9 ESCs with RNeasy mini kits (Qiagen, Hilden, Germany). Genomic DNAs were removed by DNase I (Qiagen) incubation. 0.5 μg total RNAs were then reverse transcribed into cDNA using iScript reverse transcription supermix (Bio-Rad Laboratories, Hercules, CA, USA). qPCR reactions were performed with SYBR Green supermix (Bimake, Houston, TX, USA) using the ABI 7500 Fast platform (Thermo Fisher Scientific). GAPDH was used as the housekeeping gene for gene expression normalization. Data were processed with the software associated with ABI 7500. Heatmap based on the qRT-PCR data were generated using Heatmapper (Babicki et al., 2016) (www.heatmapper.ca).

### Statistical analysis

Unless specifically indicated, all experiments were performed at least three times and data were shown as mean±standard deviations (s.d.) of the mean. Statistical analysis was carried out using either two-sample *t*-test with Minitab 18, or ANOVA with Randomized Complete Block design (RCB) and LSD post hoc test with SAS 9.4. *P*<0.05 was considered to be significant.

## Supplementary Material

Supplementary information
