# Supplementary material for: NANOG and LIN28 dramatically improve human cell reprogramming by modulating LIN41 and canonical WNT activities
Source: Biol Open. 2019 Dec 5;8(12):bio047225. doi: 10.1242/bio.047225 (PMC6918770; doi:10.1242/bio.047225)

Fig. S1. The emergence of TRA-1-60+ colony in different reprogramming conditions on day 12 and 18, respectively. +G, +N, and +L represent GLIS1, NANOG, and LIN28 added separately to OSKM; +NL, +GL, +GN, and +GNL represent the respective combinations of factors with OSKM for reprogramming. Scale bar = 250 µm.

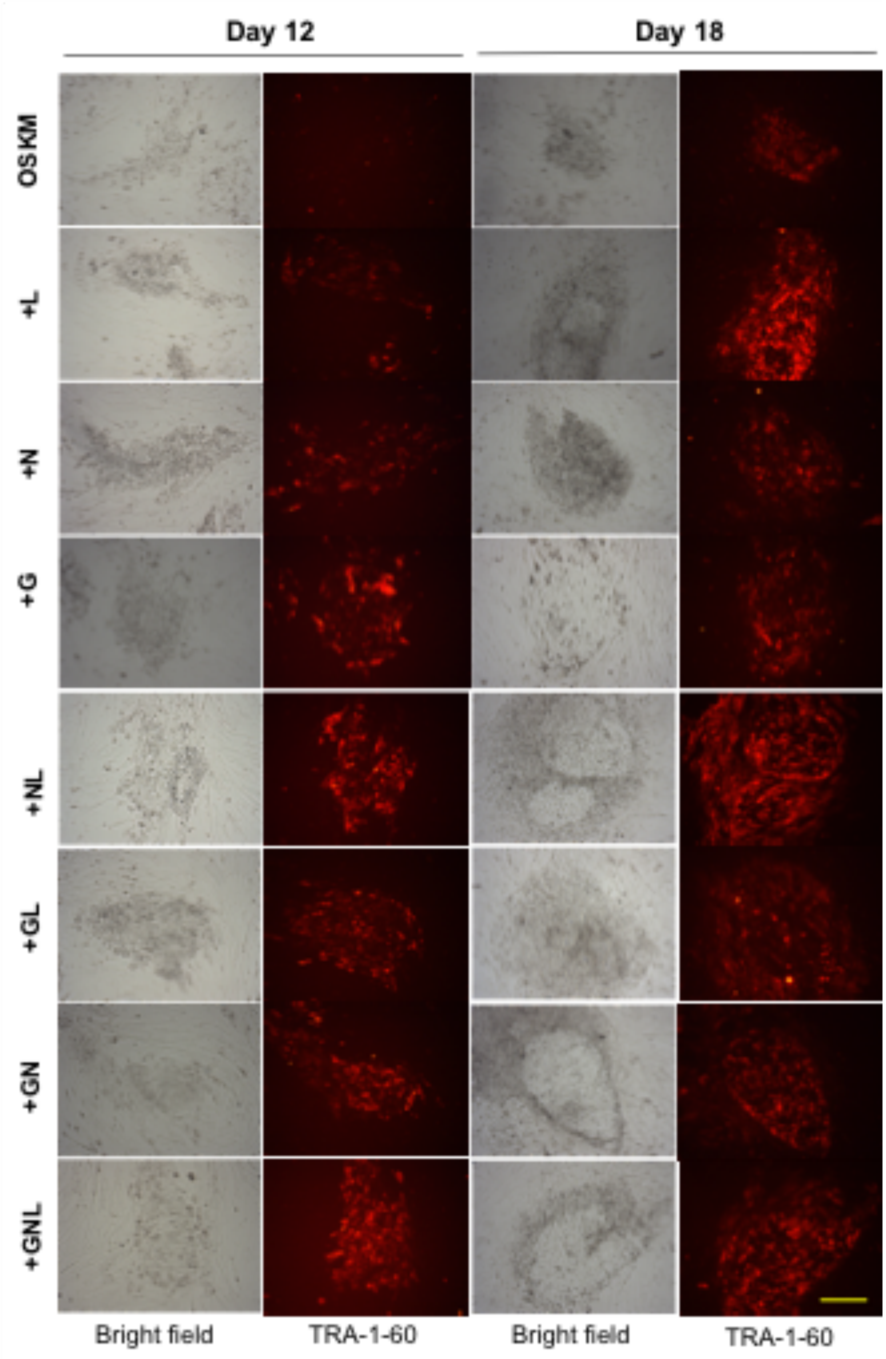


Biology Open • Supplementary information

Fig. S2. Pluripotent gene expressions in OSKM and G, N, L mediated reprogramming of human MSCs on day 14. "e" represents endogenous genes, gene expressions are relative to H9-ESCs. Bars represent mean ± s.d., n = 3.

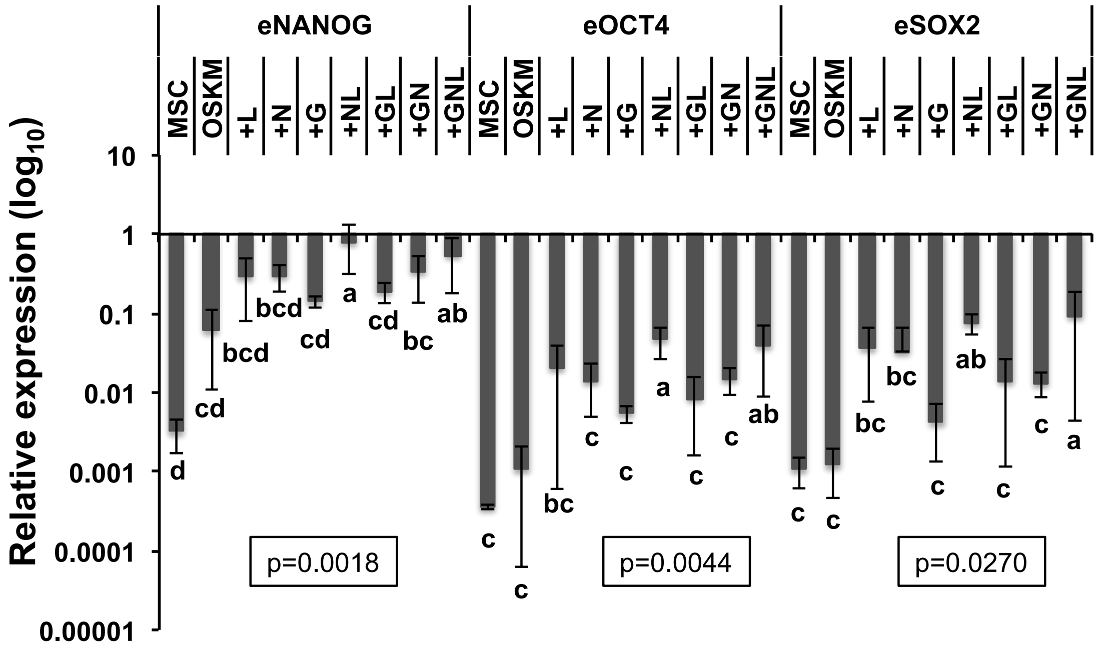


Fig. S3. Heatmap of mesenchymal gene expression in MSCs and reprogrammed cells on day 14 for different conditions. The data of each replicates are labeled by 1, 2, and 3 for different reprogramming conditions.

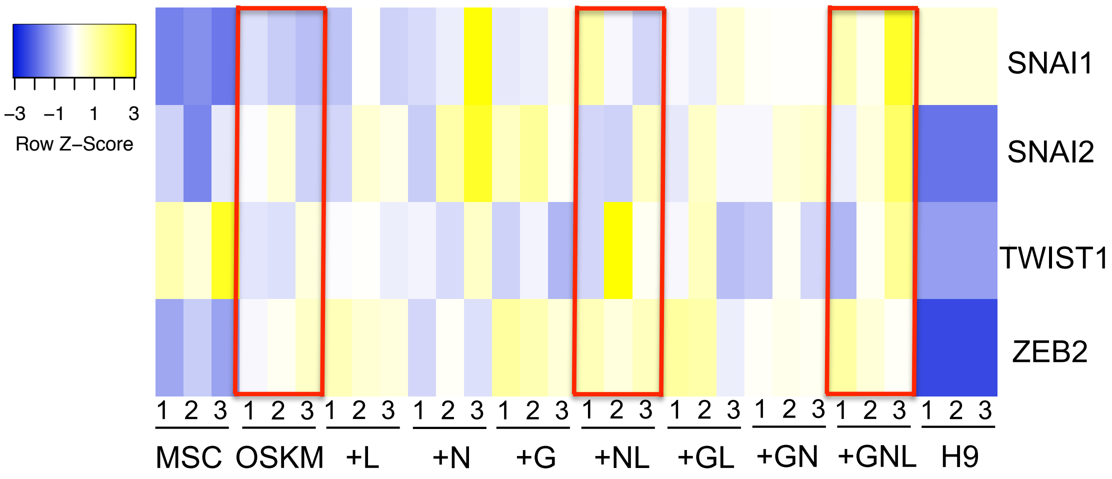


Biology Open • Supplementary information

Fig. S4. qRT-PCR analysis for total (endogenous plus transgene) LIN41 expression on reprogramming day 14. Bars represent mean ± s.d., n = 3.

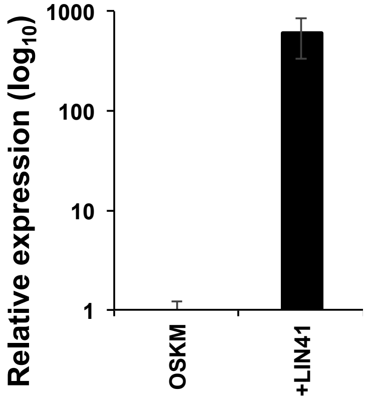


Fig. S5. Heatmap of canonical WNT ligands mRNA expression in human MSCs, 2 ($M_2O$ and $LB_2O$) iPSC lines and day 5 EBs differentiated from these iPSCs. n = 2.

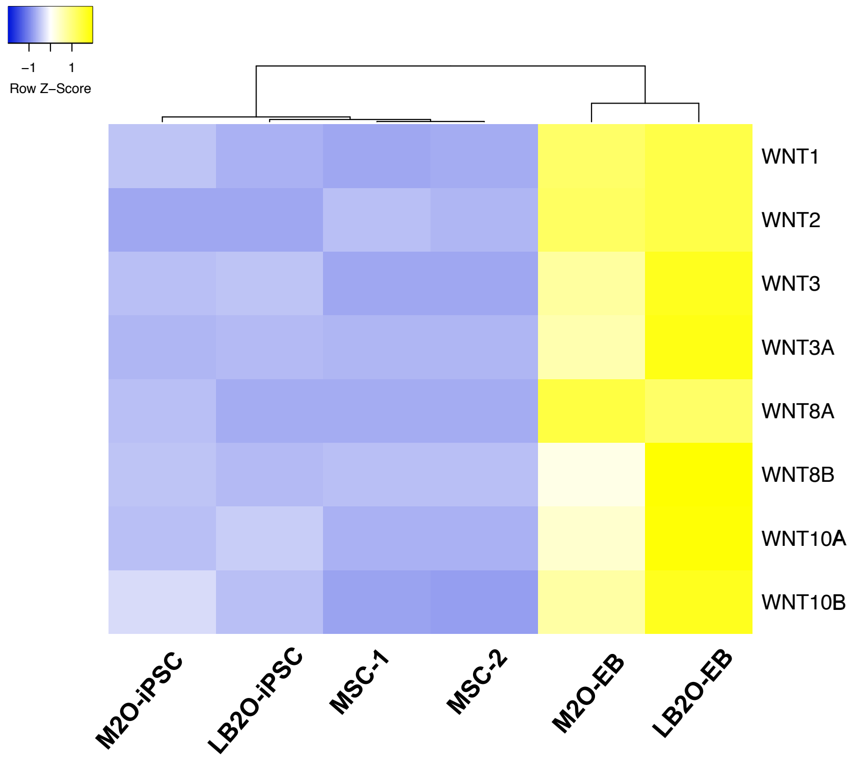


Biology Open • Supplementary information

Fig. S6. TRA-1-60+ colony numbers in OSKM and +GNLpoly mediated reprogramming conditions with or without iDOT1L on day 12. Bars represent mean ± s.d., n=3.

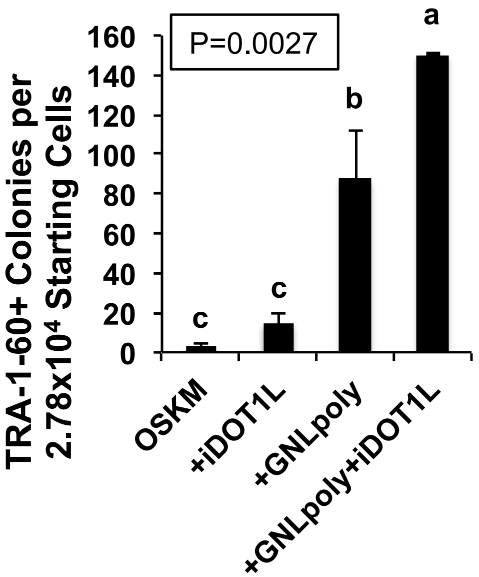


Fig. S7. Representative pictures for AP-staining of putative iPSC colonies in OSKM and +GNLpoly mediated reprogramming conditions with or without iDOT1L on day 18.

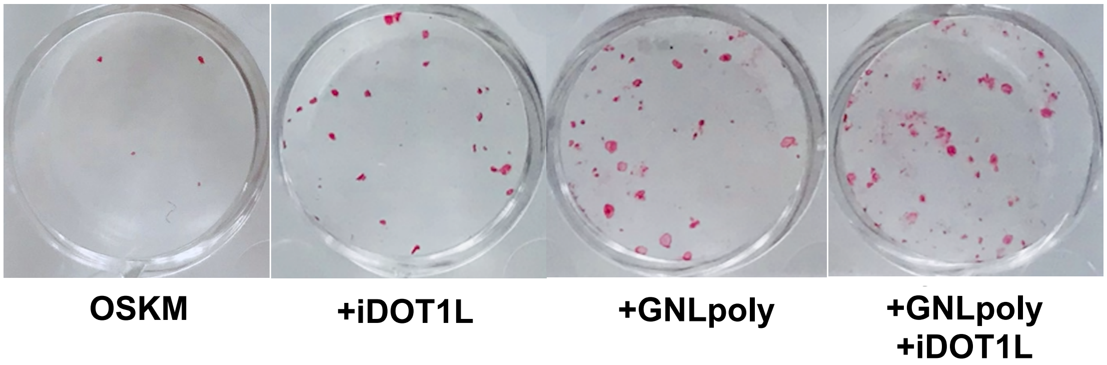


Biology Open • Supplementary information

Fig. S8. Colony morphologies in different reprogramming conditions on day 14. Representative colonies in white boxes were enlarged under each condition. Colonies in IWR1 condition had morphology more closely resembling human ESC colonies. Scale bar = 625 μm.

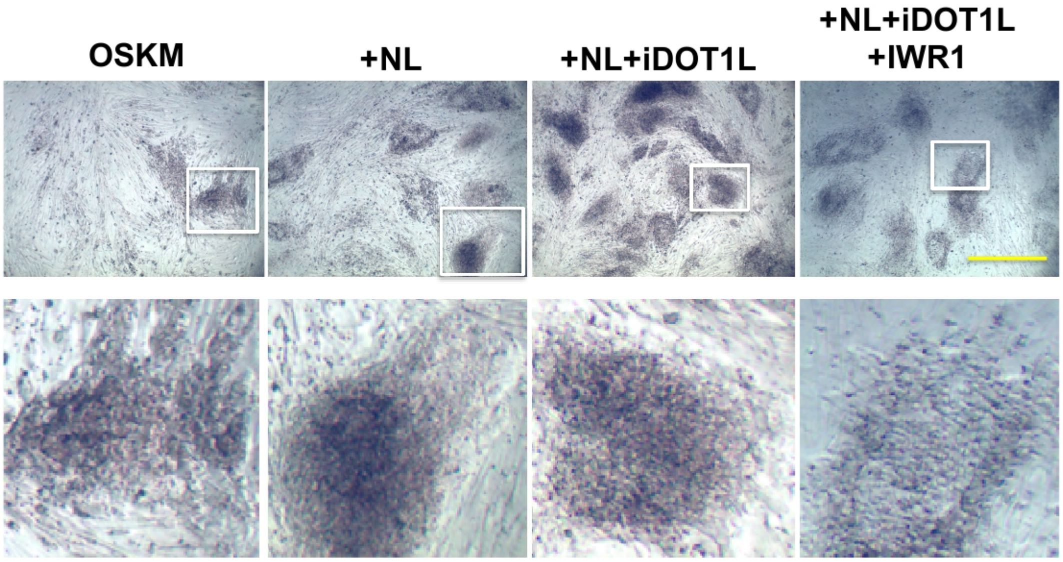


Fig. S9. Representative images of homogeneous and heterogenous TRA-1-60+ colonies in different reprogramming conditions on day 12. Scale bar = 250 μm.

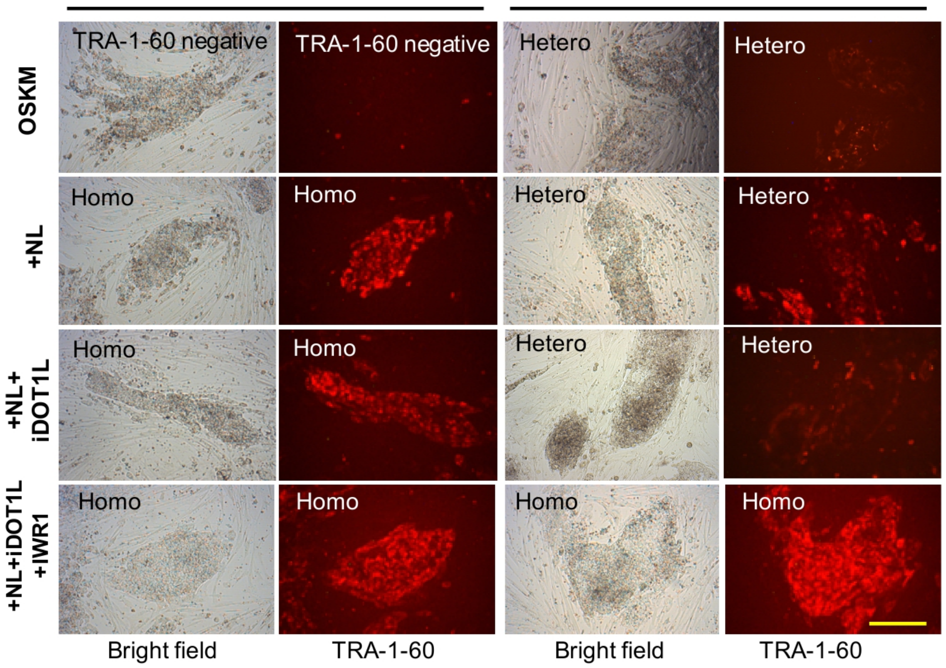


Biology Open • Supplementary information

Fig. S10. Representative pictures of putative iPSC colonies in the OSKM condition and the other reprogramming conditions stained with AP on day 18.

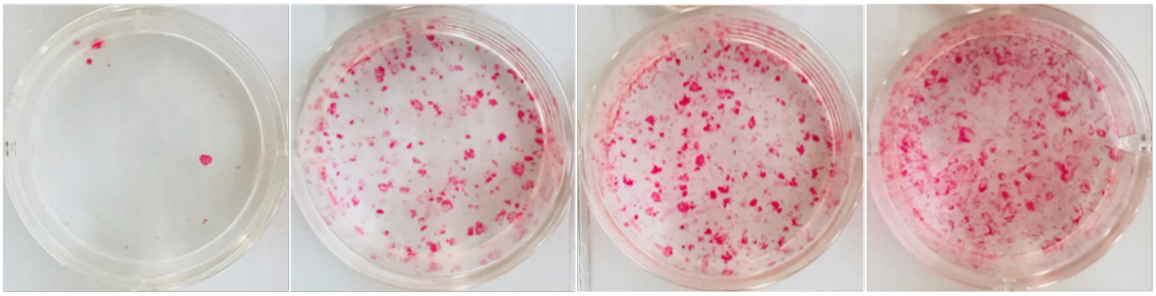


Fig. S11. qRT-PCR analysis for endogenous (e) pluripotent genes and transgene silencing as monitored by endo- (e) and total (endogenous + exogenous) gene expressions in passage 11 putative human iPSCs. Gene expressions are relative to H9-ESCs.

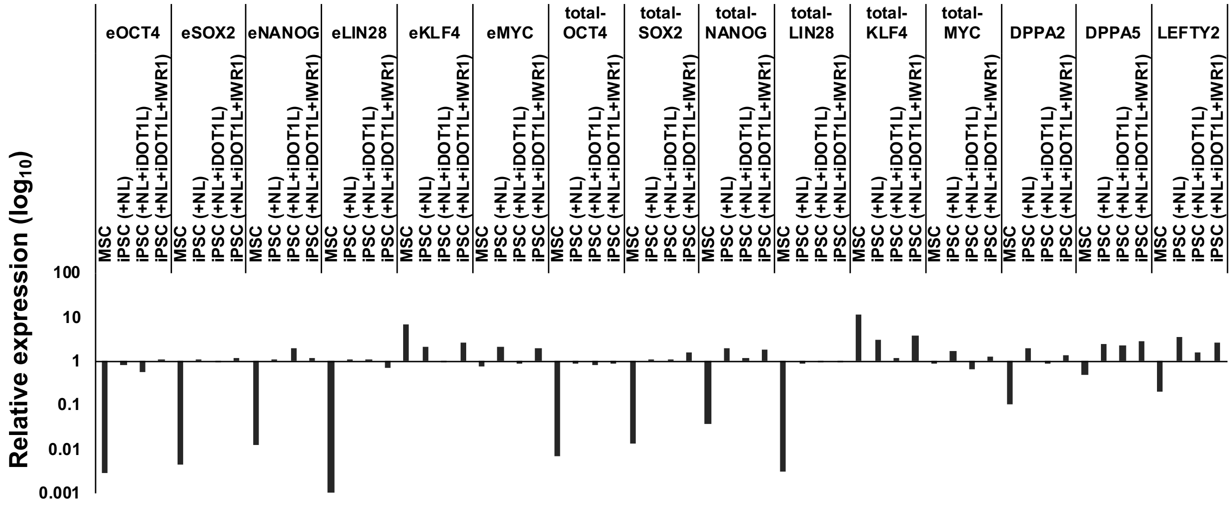


Biology Open • Supplementary information

Fig. S12. Immunostaining of pluripotent markers in passage 11 putative human iPSCs derived from +NL, +NL+iDOT1L and +NL+iDOT1L+IWR1 treated reprogramming conditions. Scale bar = 250 μm.

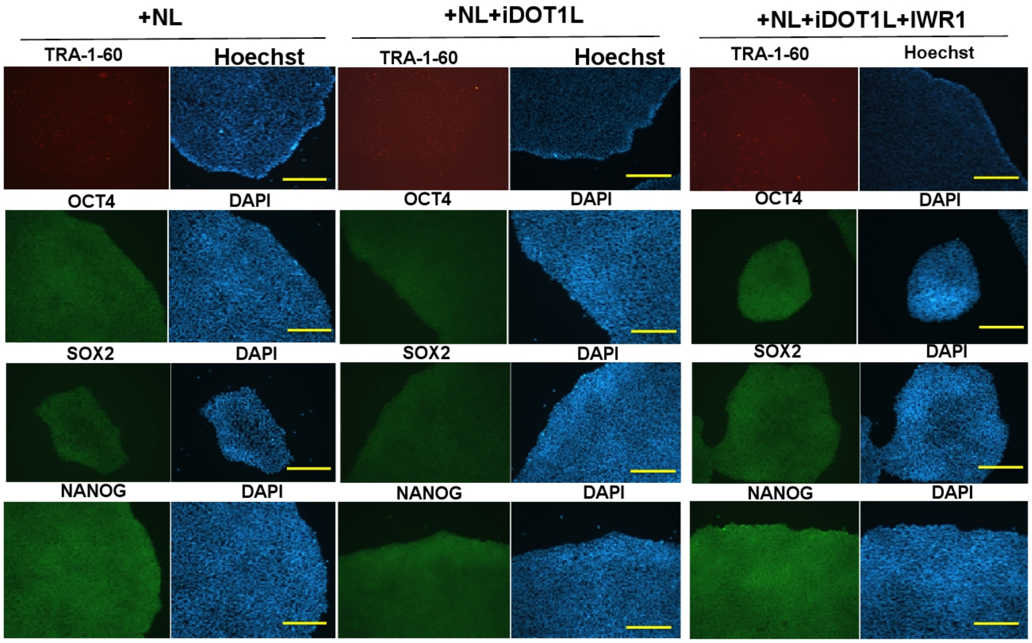


Fig. S13. Cultured putative human iPSC lines in mTeSR1 medium (left) derived from +NL, +NL+iDOT1L and +NL+iDOT1L+IWR1 treated reprogramming conditions and the respectively derived EBs on day 4 (Right). Scale bar = 250 μm.

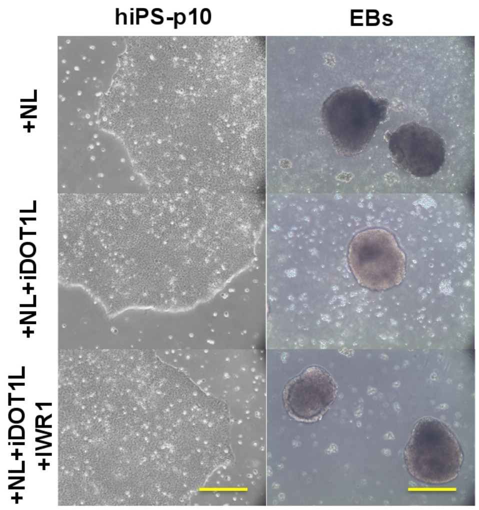


Biology Open • Supplementary information

Fig. S14. Gene expression analysis for lineage markers in day 5 EBs differentiated from the putative human iPSCs. The gene expressions in EBs were normalized to their respective parental iPSC lines.

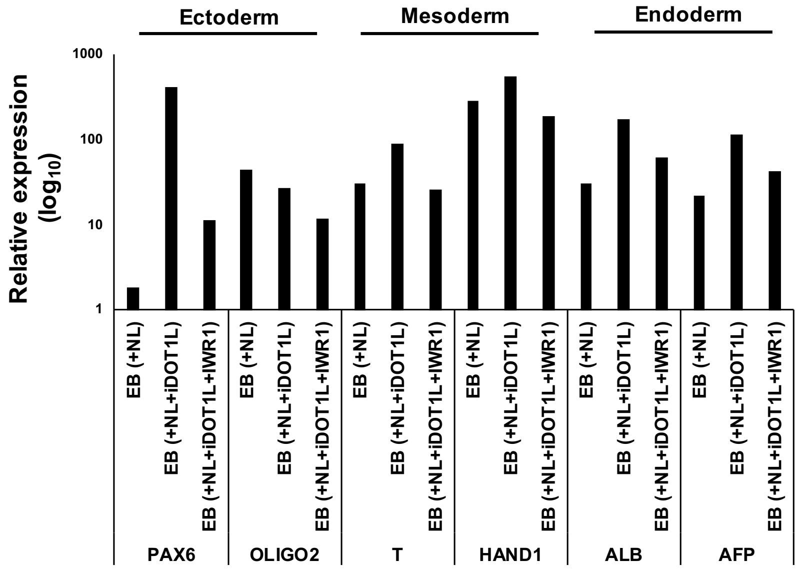


Fig. S15. Immunostaining of lineage markers for the three embryonic germ layers. Scale bar = 625 µm for OTX2 staining, and 250 µm for SMA and GATA4 staining.

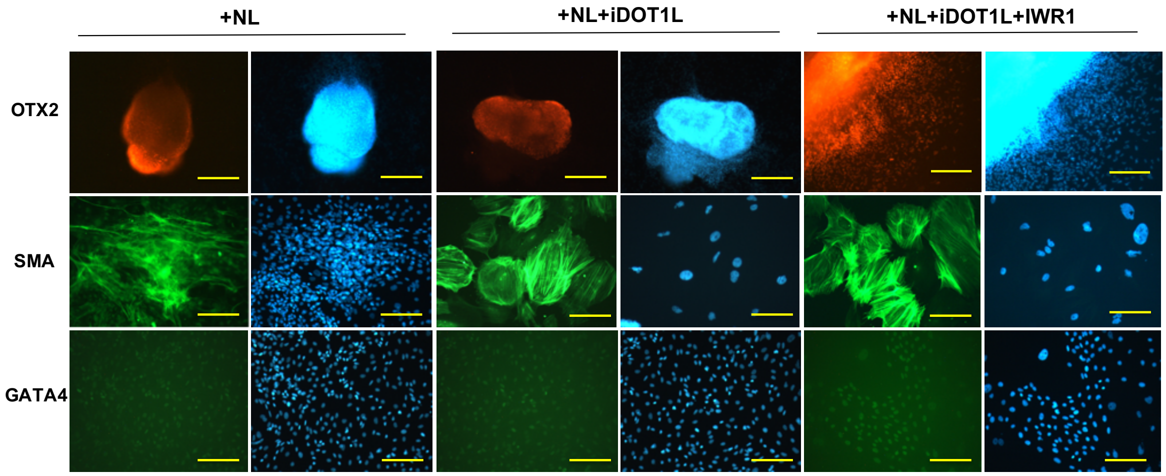


Biology Open • Supplementary information

Fig. S16. Representative pictures for AP-staining of putative iPSC colonies on day 18 for +NL+iDOT1L, +NL+iDOT1L+LIN41ΔRing, and +NL+iDOT1L with IWR1 treated from day 0 or day 7 reprogramming conditions.

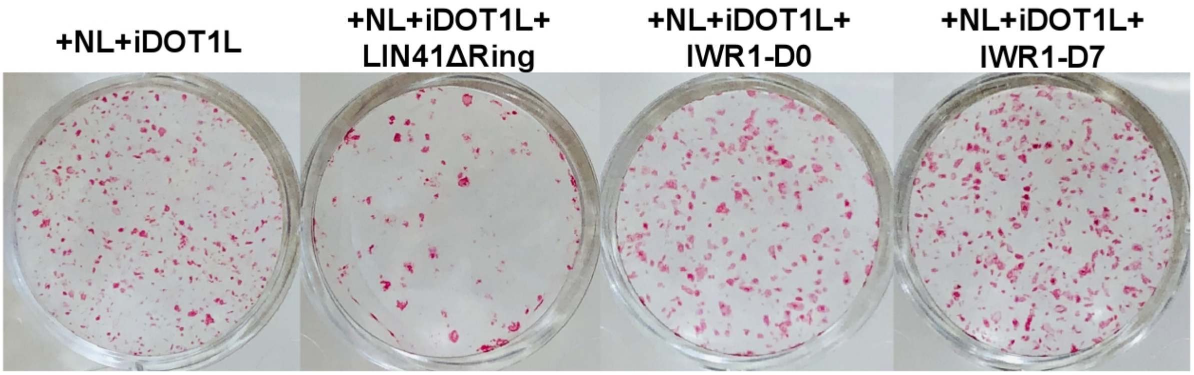


Fig. S17. Median fluorescence intensity of TRA-1-60+ cells by FACS analysis of +N+LIN41+iDOT1L condition on reprogramming day 14 with or without WNT inhibition. Bars represent mean ± s.d., n=3.

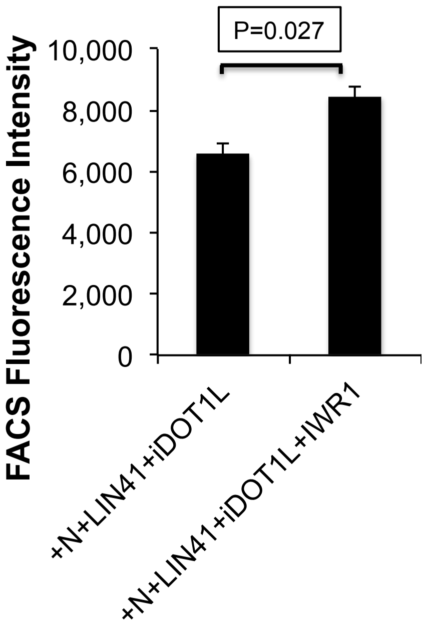

Supplement: Supplementary information [file biolopen-8-047225-s1.pdf]
